# The Alcoholic and Opium Habit

**Published:** 1889-08

**Authors:** 


### SELECTIONS.

### The Alcoholic and Opium Habit.

At the meeting of the Illinois State Medical Society, May 21, Dr. Charles Warrington Earle, of Chicago, read an address on "The Duties and Responsibilities of the Medical Profession regarding Alcoholic and Opium Inebriation." Dr. Earle had had eighteen years' experience in a reformatory institution, and had treated upwards of ten thousand cases during that time, consequently he was well qualified to deal with the subject intelligently and instructively. He asserted that the medical profession was not exercising its influence in the right direction in regard to temperance; many prescribe alcohol and morphine without limit, many victims take their first dose from a physician, and too many physicians themselves become addicted to the habits. Dr. Earle declared that alcoholism is not a disease. Ninety-nine out of a hundred drunkards show no symptoms of disease. A man can cure himself of the habit by exercise of the will, unaided by medicine, but no disease can be cured in that way. No physical cause compels a man to become a slave. Ninety-nine out of one hundred men can reform if they will. Reformation requires a cultivation of the moral sense, a strengthening of the will, a cutting off of all evil associations and all bad habits, and total abstinence from the use of liquor. A man should carefully avoid all temptation. It would not do for him to attempt to drink in moderation. It is true that alcoholism may produce changes in every tissue and bring about all manner of diseases, but it is not a disease itself; and a man who has drunk ten, twenty, or thirty years may stop as easily (*sic*) as a man who has drunk only three months. Dr. Earle said that neither the alcoholic nor the morphine habit is hereditary, and he quoted figures to prove it from his own experience. Idleness and want of government are, he said, more prolific causes of drunkenness. The most marked results of inebriety are not physical, but mental and moral. They are not productive of disease to the extent we are led to believe. Physicians should be temperate for their own sakes, and also to set examples for others. They should be more careful how they prescribe alcohol and morphine. The last is the more seductive, and physicians are responsible for the morphine habit in a majority of cases. It should be prescribed only in perilous cases, and not at all for trivial complaints, as is too common. Dr. Earle advised that the young be educated to avoid the habits in question; that work be prosecuted among those who earnestly desire reformation, and that legislation be sought for the incorrigible persons who cannot be reformed. For the latter he recommended state guardianship and two years on a farm.—*Peoria Medical Monthly, May*, 1889.

